# Factors associated with pain in nonsurgically treated rotator cuff tears -A study with magnetic resonance imaging

**DOI:** 10.1186/s13018-019-1178-x

**Published:** 2019-05-14

**Authors:** Yoshihiro Nakamura, Shin Yokoya, Yohei Harada, Mitsuo Ochi, Nobuo Adachi

**Affiliations:** 1 Department of Orthopaedic Surgery, Chugoku Rosai Hospital, 1-5-1, Tagaya, Hiro, Kure City, Hiroshima, 737-0193 Japan; 2 Department of Orthopaedic Surgery, Hiroshima University, 1-2-3 Kasumi, Minami-ku, Hiroshima City, Hiroshima, 734-8551 Japan; 3 Department of Orthopaedic Surgery, Aichi Medical University, 1-1 Yazakokarimata, Nagakute City, Aichi 480-1195 Japan; 4 Hiroshima University, 1-4-1 Kagamiyama, Higashi-hiroshima City, Hiroshima, 739-8527 Japan; 5 Department of Orthopaedic Surgery, Hiroshima University, 1-2-3 Kasumi, Minami-ku, Hiroshima City, Hiroshima, 734-8551 Japan

**Keywords:** Rotator cuff tear, Nonsurgical treatment, Magnetic resonance imaging, Pain, Subscapularis tendon tear

## Abstract

**Background:**

In rotator cuff tears, some cases become asymptomatic with nonsurgical treatment, others remain symptomatic. The purpose of this study was to identify factors associated with pain in nonsurgically treated rotator cuff tears using magnetic resonance imaging (MRI).

**Methods:**

In total, 108 shoulders diagnosed with supraspinatus (SSP) tendon tears using MRI were nonsurgically treated, and MRI was repeated after more than a year. The patients were divided into pain or improvement group according to whether the pain persisted or disappeared. Bursal fluid accumulation; SSP tendon retraction; subscapularis (SSC) tendon tears; infraspinatus (ISP) tendon tears; and Goutallier classification into SSC, SSP, and ISP were included as evaluation factors. Predictive factors for persistent pain on initial MRI and factors associated with persisting pain after nonsurgical treatment on repeat MRI were statistically analyzed using multivariate logistic regression analysis.

**Results:**

The improvement group showed a significant decrease in bursal fluid accumulation compared with the pain group (p < 0.01). SSC tendon tears (OR, 4.42; 95% CI, 1.16–16.9; P = 0.03) on initial MRI were significantly associated with persistent pain. Bursal fluid accumulation (OR, 2.44; 95% CI, 1.18–5.07; P = 0.02) and SSC tendon tears (OR, 2.25; 95% CI, 1.15–4.39; P = 0.02) on repeat MRI were significantly associated with persistent pain.

**Conclusions:**

Bursal fluid accumulation decreased when pain improved. The involvement of SSC tendon tears can serve as a predictive factor for persistent pain. Pain may persist although patients with rotator cuff tears including SSC tendon tears are nonsurgically treated.

**Level of evidence:**

Level IV case-control study

## Background

A rotator cuff tear is a common cause of pain and disability of the shoulder among adults. Some rotator cuff tears are symptomatic, whereas others are asymptomatic [1, 2]. Treatment strategy for rotator cuff tears remains unclear [3]. Because nonsurgical treatment often improves the pain and range of motion in patients with symptomatic rotator cuff tears [4], it is typically preferred as the first-line treatment [5, 6]. Conversely, surgical treatment is occasionally required when symptoms do not improve. Success rates of nonsurgical treatment have been reported to vary widely, ranging 33–82% [6–10]. Itoi et al. reported that patients with complete rotator cuff tears, and who had been treated conservatively, exhibited unsatisfactory results with limited active abduction angle and muscle weakness on abduction on first examination [8]. Tanaka et al. reported that an intact intramuscular tendon of the supraspinatus (SSP), showing little or no atrophy of the SSP muscle, with negative impingement signs, and with preserved motion in external rotation are factors related to the successful outcome of conservative treatments [6]. Boorman et al. reported that the rotator cuff quality-of-life index was predictive of the outcome of non-operative treatment in patients with a chronic full-thickness rotator cuff tear [11]. Dunn et al. reported that patient expectations regarding the role of rehabilitation were the strongest predictor of surgery and other factors associated with surgery were higher activity level and not smoking [12]. However, a failure of nonsurgical treatment was defined if the patient elected to have surgery after nonsurgical treatment in most previous studies. There may be a potential selection bias because some patients do not want to undergo surgery whereas others want to undergo surgery and failure of nonsurgical treatment is determined relatively early in previous studies. It is unclear about the difference between cases that become asymptomatic with nonsurgical treatment and cases that nonsurgical treatment is continued as symptomatic. Although most rotator cuff tears occur in the SSP tendon, other tendons of the rotator cuff may also be torn. In addition, the degree of fatty infiltration of rotator cuff muscles varies. The effect of nonsurgical treatments may depend on the severity of the rotator cuff tears. Therefore, the purpose of this study was to identify the factors associated with persisted pain in nonsurgically treated rotator cuff tears using magnetic resonance imaging (MRI).

## Methods

### Study population

From 2010 to 2014, all symptomatic patients diagnosed with full-thickness SSP tendon tears, regardless of whether infraspinatus (ISP) and subscapularis (SSC) tendon tears existed or not on MRI, were nonsurgically treated using steroids or hyaluronic acid injections and anti-inflammatory analgesics. Patients were recommended for surgery when symptoms persisted after nonsurgical treatment for at least 3 months. Patients who rejected to undergoing surgery were continued to be nonsurgically treated. The treatment was terminated if subjective symptoms disappeared by nonsurgical treatment, and the patients were regularly followed-up. Patients were reexamined using MRI at more than 1 year after the initial MRI. Patients who underwent surgery; patients who could not be followed-up; and patients with a history of surgery, fracture, osteoarthritis, or purulent arthritis of ipsilateral shoulder joints; patients with rheumatoid arthritis; and patients suspected to have neurological disease or cervical spine disease, such as cervical spondylotic amyotrophy, were excluded from this study. In total, 97 patients (108 shoulders), including 49 males (55 shoulders) and 48 females 53 shoulders), with a mean age of 68.9 ± 8.1 (range, 44–84) years, were enrolled. Mean follow-up period was 22.6 ± 12.4 (range, 12–54) months. The patients were divided into pain group and improvement group. Pain group consisted of patients that pain persisted until repeat MRI and required continued nonsurgical treatments such as steroids, hyaluronic acid injections, and/or anti-inflammatory analgesics. Improvement group consisted of patients that became asymptomatic and the treatment was terminated before repeat MRI. Finally, 63 patients (67 shoulders; mean age, 69.4 ± 8.2 years; males, 32 shoulders; females, 35 shoulders; mean follow-up period, 21.4 ± 10.8 months) were included in the pain group and 38 patients (41 shoulders; mean age, 68.1 ± 8.1 years; males, 23 shoulders; females, 18 shoulders; mean follow-up period, 24.9 ± 14.5 months) were included in the improvement group.

### MRI

MRI examinations were performed using a 1.5 Tesla scanner (Sigma; GE Medical Systems, Milwaukee, WI, USA or EXCELART Vantage; Toshiba Medical Systems, Tochigi, Japan). The patients were rested in a supine position with the affected arm toward the side of the body, with the palm of the hands facing upward, for the imaging of the shoulder joints. The imaging protocol included the following sequences (Sigma/EXCELART Vantage): oblique coronal T2-weighted imaging [repetition time (TR), 5000/3200 ms; echo time (TE), 90/90 ms; slice thickness, 4.5/4 mm; slice gap, 0.5/0.8 mm; field of view (FOV), 18/20 cm; matrix, 320 × 288/240 × 240 pixels; and echo train length (ETL), 18/23]; axial T2-weighted imaging [TR, 4500/3200 ms; TE, 90/90 ms; slice thickness, 4.5/4 mm; slice gap, 0.5/0.8 mm; FOV, 18/20 cm; matrix, 320 × 256/240 × 240 pixels; and ETL: 20/23]; oblique sagittal T1-weighted imaging [TR, 850/700 ms; TE, 10.2/15 ms; slice thickness, 4.5/4 mm; slice gap, 0.5/0.8 mm; FOV, 18/20 cm; matrix, 288 × 192/224 × 320 pixels; and ETL, 2/5]; and oblique sagittal T2-weighted imaging [TR, 5000/4200 ms; TE, 90/90 ms; slice thickness, 4.5/3 mm; slice gap, 0.5/0 mm; FOV, 18/14 cm; matrix, 320 × 256/384 × 269 pixels; and ETL, 20/12]. The same MRI scanner that was initially used was used for the repeat MRI.

### Evaluation

Bursal fluid accumulation; SSP tendon retraction; SSC tendon tears; ISP tendon tears; and Goutallier classification in SSC, SSP, and ISP were included as evaluation factors. Subcoracoid–subacromial–subdeltoid bursal fluid accumulation was evaluated. The volume of fluid was graded as follows: grade 0, bursal fluid not detected; grade 1, the thickness of bursal fluid < 5 mm; and grade 2, the thickness of bursal fluid ≥ 5 mm. SSP tendon tear retraction was measured from the torn SSP tendon stump to the lateral aspect of the greater tuberosity on an oblique coronal MRI (T2-weighted imaging). SSC tendon tears were diagnosed on the basis of the axial and oblique sagittal T2-weighted MRI. A partial tear was diagnosed if high signal intensity was detected in the most proximal portion of the SSC tendon but did not involve the full thickness of the tendon. A complete tear was diagnosed if a detachment of SSC tendon insertion was detected. Cuff tears spreading to the middle facet on the oblique sagittal MRI (T2-weighted imaging) were defined as ISP tendon tears. The fatty infiltration of the SSC, SSP, and ISP muscles was assessed on an oblique sagittal MRI (T1-weighted imaging) according to the modified Goutallier classification using a slice of the outermost edge touching the spine and the body of the scapula [13, 14]. Changes in these factors were analyzed in each group between initial MRI and repeat MRI.

### Statistical methods

Chi-squared test was used to evaluate differences in the decreasing rate in bursal fluid accumulation and the progression rates of SSC, ISP tendon tears, and Goutallier classification in SSC, SSP, and ISP between the two groups. Mann–Whitney *U* tests were used to investigate differences in the progression of SSP tendon retraction between the two groups. Logistic regression analysis was performed to investigate predictive factors for persistent pain on the initial MRI and factors associated with persisting pain after nonsurgical treatment on the repeat MRI. Odds ratios (ORs) and 95% confidence intervals (CIs) were calculated. From the univariate logistic regression analysis, the variables that had a *P* value of < 0.05 were considered as potential variables. Multivariate logistic regression was performed in the next phase. *A P* value of < 0.05 was considered statistically significant.

## Results

### Changes in the factors

The changes in each factor are shown in Table 1. As bursal fluid accumulated in the pain group, one of four grade 0 shoulders increased to grade 1. Of the 34 shoulders that were grade 1, 4 shoulders decreased to grade 0 and 8 shoulders increased to grade 2. Six of the 29 grade 2 shoulders decreased to grade 1. As the bursal fluid in improvement group, of 6 shoulders that were grade 0, 1 shoulder increased to grade 1 and 1 shoulder increased to grade 2. Five of 20 grade 1 shoulders decreased to grade 0. Of 15 shoulders that were grade 2, 4 shoulders decreased to grade 0 and 5 shoulders decreased to grade 1. The improvement group showed a significant decrease compared with the pain group in bursal fluid accumulation (*P* < 0.01). SSP tendon tear retraction progressed from 24.4 ± 13.5 mm to 28.5 ± 14.1 mm in pain group and from 17.1 ± 13.8 mm to 21.2 ± 14.4 mm in improvement group. The average progression of SSP tendon tear retraction was 4.1 ± 5.4 mm in the pain group and 4.1 ± 5.2 mm in the improvement group, and there was no significant difference between the two groups (*P* = 0.90). With regard to SSC tendon tears, in the pain group, the progression rate of SSC tendon tears was 11.9%. In the improvement group, the progression rate of SSC tendon tears was 2.4%. There was no significant difference in progression rate of SSC tendon tears between the two groups (*P* = 0.08). For ISP tendon tears, the progression rate of ISP tendon tears was 11.9% in the pain group, while in the improvement the progression rate was 7.3%. There was no significant difference in progression rate of ISP tendon tears between the two groups (*P* = 0.44). In the Goutallier classification in SSC, the progression rate of the Goutallier classification for SSC was 3.0% in the pain group, while in the improvement group it was 7.3%. No significant differences were seen for this parameter between the two groups (*P* = 0.30). The progression rate according to the Goutallier classification for SSP in the pain group was 14.9%, while in the improvement group it was 14.6%. No significant difference in progression rate of Goutallier classification in SSP between the two groups was noted (*P* = 0.97). With regards to the Goutallier classification for ISP, in the pain group, the progression rate was 17.9%, and in the improvement group, it was 12.2%. No significant differences in the progression rate of the Goutallier classification in ISP between the two groups was noted (*P* = 0.43).

**Table 1:** Changes in factors

	Pain group		Improvement group	
	Initial MRI	Repeat MRI	Initial MRI	Repeat MRI
Bursal fluid accumulation				
Grade 0	4	7	6	13
Grade 1	34	29	20	21
Grade 2	29	31	15	7
SSP retraction				
(mm)	24.4 ± 13.5	28.5 ± 14.1	17.1 ± 13.8	21.2 ± 14.4
SSC tendon tears				
No tears	26	20	29	28
Partial tears	19	23	7	8
Complete tears	22	29	5	5
ISP tendon tears				
No tears	41	33	33	30
Tears	26	34	8	11
Goutallier classification in SSC				
Grade 0	24	22	26	26
Grade 1	21	23	8	7
Grade 2	10	10	3	3
Grade 3	4	4	2	1
Grade 4	8	8	2	4
Goutallier classification in SSP				
Grade 0	0	0	1	0
Grade 1	15	14	15	14
Grade 2	31	28	21	20
Grade 3	9	12	1	3
Grade 4	12	13	3	4
Goutallier classification in ISP				
Grade 0	7	5	10	10
Grade 1	33	30	24	20
Grade 2	9	9	3	6
Grade 3	4	6	0	0
Grade 4	14	17	4	5

*ISP*, infraspinatus; *SSC*, subscapularis; *SSP*, supraspinatus

### Predictive factor for persistent pain in initial MRI

For multivariate logistic regression analysis to investigate predictive factor for persistent pain in initial MRI, variables with *P* < 0.05 in univariate models were selected (Table 2). Therefore, SSP tendon retraction, SSC tendon tears, ISP tendon tears, and Goutallier classification in SSC, SSP, and ISP were selected. On the multivariate logistic regression analysis, SSC tendon tears (OR, 4.48; 95% CI, 1.14–16.0; *P* = 0.03) on initial MRI were considered as predictive factor for persistent pain (Table 3).

**Table 2:** Univariate logistic regression analysis in initial MRI

Predictive factor for persistent pain	OR	95% CI	*P* value
1. Bursal fluid accumulation	1.47	0.79–2.72	0.224
2. SSP tendon tear retraction	1.04	1.01–1.08	0.010*
3. SSC tendon tears	2.34	1.36–4.02	0.002*
4. ISP tendon tears	2.62	1.05–6.53	0.040*
5. Goutallier classification in SSC	1.50	1.05–2.15	0.026*
6. Goutallier classification in SSP	1.83	1.15–2.90	0.011*
7. Goutallier classification in ISP	1.58	1.10–2.27	0.013*

CI, confidence interval**;** ISP, infraspinatus; OR, odds ratio; SSC, subscapularis; SSP, supraspinatus

*P value < 0.05

**Table 3:** Multivariate logistic regression analysis in initial MRI

Predictive factor for persistent pain	OR	95% CI	*P* value
2. SSP tendon tear retraction	1.00	0.96–1.05	0.87
3. SSC tendon tears	4.28	1.14–16.0	0.03*
4. ISP tendon tears	1.37	0.31–6.09	0.68
5. Goutallier classification in SSC	0.54	0.24–1.23	0.14
6. Goutallier classification in SSP	1.33	0.64–2.75	0.45
7. Goutallier classification in ISP	1.05	0.56–1.99	0.87

*CI*, confidence interval; *ISP*, infraspinatus; *OR*, odds ratio; *SSC*, subscapularis; *SSP*, supraspinatus

*P value < 0.05

### Factor associated with persisting pain after nonsurgical treatment in repeat MRI

Multivariate logistic regression analysis was employed to investigate the factors associated with persisting pain after the nonsurgical treatment in repeat MRI. Variables with *P* < 0.05 in univariate models were selected (Table 4). Therefore, bursal fluid, SSP tendon retraction, SSC tendon tears, ISP tendon tears and Goutallier classification in SSP, and ISP were selected. Multivariate logistic regression analysis found that bursal fluid (OR, 2.80; 95% CI, 1.40–5.61; *P* = 0.04) and SSC tendon tears (OR, 2.37; 95% CI, 1.21–4.64; *P* = 0.01) on repeat MRI were significantly associated with persistent pain (Table 5).

**Table 4:** Univariate logistic regression analysis in repeat MRI

Factor associated with persisting pain	OR	95% CI	*P* value
1. Bursal fluid accumulation	3.17	1.69–5.94	0.0003*
2. SSP tendon tear retraction	1.04	1.01–1.07	0.013*
3. SSC tendon tears	2.81	1.60–4.92	0.0003*
4. ISP tendon tears	2.81	1.21–6.51	0.016*
5. Goutallier classification in SSC	1.39	1.00–1.94	0.053
6. Goutallier classification in SSP	1.75	1.13–2.69	0.012*
7. Goutallier classification in ISP	1.57	1.12–2.20	0.0082*

*CI*, confidence interval; *ISP*, infraspinatus; *OR*, odds ratio; *SSC*, subscapularis; *SSP*, supraspinatus

*P value < 0.05

**Table 5:** Multivariate logistic regression analysis in repeat MRI

Factor associated with persisting pain	OR	95% CI	*P* value
1. Bursal fluid accumulation	2.80	1.40–5.61	0.04*
2. SSP tendon tear retraction	0.99	0.94–1.04	0.75
3. SSC tendon tears	2.37	1.21–4.64	0.01*
4. ISP tendon tears	1.78	0.44–7.18	0.42
6. Goutallier classification in SSP	0.83	0.37–1.82	0.64
7. Goutallier classification in ISP	1.13	0.64–2.00	0.69

*CI*, confidence interval; *ISP*, infraspinatus; *OR*, odds ratio; *SSC*, subscapularis; *SSP*, supraspinatus

*P value < 0.05

## Discussion

There are several reports of predictors of outcome of nonsurgical treatment for rotator cuff tears [6, 8, 11, 12]. However, nonsurgical treatment outcome was defined as a success if surgical treatment was not required in most previous studies. Strengths of our study include re-assessing via MRI patients who did not have surgery with more than a year of follow-up, and we compared patients that became asymptomatic by nonsurgical treatment with patients that pain persisted and nonsurgical treatment is continued.

There are a few reports comparing MRI findings in symptomatic and asymptomatic rotator cuff tears. Some reports showed that there was no difference [15, 16], others showed that there were significant associations between symptoms and tear size, positive tangent sign, and fatty degeneration [17]. It was often reported that symptoms of pain do not correlate with rotator cuff tear severity [18–20]. However, there are currently no reports to examine SSC tendon tears in previous studies, apart from a study conducted by Harris et al. who reported that involvement of the SSP, ISP, and SSC tendons was associated with a decreased American Shoulder and Elbow Surgeons score comparing isolated SSP tears in patients with symptomatic and traumatic full-thickness rotator cuff tears who are enrolled in a structured physical therapy program [19]. In our study, we evaluated factors associated with pain in nonsurgically treated SSP tendon tears by MRI. Bursal fluid accumulation, SSP tendon retraction, SSC tendon tears, ISP tendon tears, and Goutallier classification in SSC, SSP, and ISP were included as evaluation factors in the multivariate logistic regression analysis. Consequentially, bursal fluid decreased when pain improved. This suggested the subcoracoid–subacromial–subdeltoid bursal fluid was associated with the presence of pain. However, bursal fluid could not be a predictive factor for persistent pain in the initial MRI. On the other hand, rotator cuff tears with the involvement of SSC tendon tears on initial MRI were considered as predictive factors for persistent pain.

The balance between the SSC muscle and the ISP and teres minor muscles is often referred to as the rotator cuff “transversal force couple.” It generates stability in the glenohumeral joint by an antagonistic balance between the forces exerted in an anterior and posterior part [21]. This anterior–posterior force balance between the SSC and the ISP, and teres minor causes compression of the humeral head into the glenoid fossa in patients with isolated SSP tendon tears [22–24]. Even when there are irreparable rotator cuff tears, shoulder function can be preserved if there is a balance between important force couples [25, 26]. Therefore, damage of transversal force couples is suggested as the cause of the correlation between SSC tendon tears and persistent pain. On the other hand, ISP tendon tears were not a factor associated with persistent pain in this study. This may be due to compensation by teres minor muscles. It has been reported that progression of ISP muscle atrophy induces compensatory hypertrophy of the teres minor muscles [27, 28].

The SSC has other crucial functions in the shoulder joint. The SSC stabilizes the long head of the biceps tendon. It was reported that most SSC tendon tears occurred on the most superior part and the articular side of the attachment of the humerus [29, 30]. Ide et al. reported that the SSC insertion to the humerus consisted of a proximal tendinous part and a distal muscular part in anatomical study [31]. Arai et al. reported that anatomically, the most superior part of the SSC tendon was attached to the upper margin of the lesser tuberosity and extended as a thin tendinous slip to the fovea capitis of the humerus [32]. The trochlea-like structure was composed of the superior-most insertion, the tendinous slip, and the lateral portion of the cranial part of intramuscular tendons supporting the long head of the biceps tendon. SSC tendon tears, involving injuries of the trochlea-like structure, causes instability and pathology of the long head of the biceps tendon [33, 34]. SSC tendon tears may induce lesion of the long head of biceps tendon and cause pain.

For the reasons stated above, in this study, SSC tendon tears may be associated with persistent pain for nonsurgically treated rotator cuff tears. Arthroscopic repair with use of a suture anchor technique has been reported to be a safe and effective procedure for the treatment of combined rotator cuff tears involving the SSC tendon [35]. It has also been demonstrated that improvement in functional outcome after arthroscopic repair of an SSC tendon tear is maintained long-term [36]. Therefore, if no effect is seen from nonsurgical treatment for rotator cuff tears with the involvement of SSC tendons, surgical repair can be considered.

## Limitations

There were several limitations to this study. First, the number of cases included is small. Second, nonsurgical treatment was not standardized as the frequency of injection varied. Third, there is no scale for pain evaluation. Conditions requiring continued nonsurgical treatments such as steroids, hyaluronic acid injections, and/or anti-inflammatory analgesics were defined as persistent pain. Fourth, interpretations of MRI are not always accurate [37, 38]. However, Adam et al. state that preoperative shoulder MRI scans interpreted by orthopedic surgeons via a systematic approach resulted in improved accuracy in diagnosing SSC tendon tears [39]. Yoshimura et al. focused on the anatomical structure of SSC and evaluated MRI findings of the most proximal portion of the SSC tendon on axial and oblique sagittal T2-weighted images [40]. Based on their report, SSC tendon tears were evaluated on MRI in this study.

## Conclusions

We evaluated the factors associated with pain in nonsurgically treated rotator cuff tears by MRI. Bursal fluid accumulation decreased when pain improved, but the bursal fluid is not a reliable predictive factor for persistent pain in initial MRI. On the other hand, SSC tendon tears on initial and repeat MRI were significantly associated with persistent pain. Therefore, the involvement of SSC tendon tears may serve as a predictive factor in persistent pain. SSC function may be important since SSC contributes to “transversal force couple” and stabilizes the long head of the biceps tendon. When patients with rotator cuff tears including SSC tendon tears are treated nonsurgically, attention to the possibility of persistent pain should be given.

## Abbreviations

CIs: Confidence intervals
ETL: Echo train length
FOV: Field of view
ISP: Infraspinatus
MRI: Magnetic resonance imaging
ORs: Odds ratios
SSC: Subscapularis
SSP: Supraspinatus
TE: Echo time
TR: Repetition time
